# *RELA* governs a network of islet-specific metabolic genes necessary for beta cell function

**DOI:** 10.1007/s00125-023-05931-6

**Published:** 2023-06-14

**Authors:** Nathan W. Zammit, Ying Ying Wong, Stacey N. Walters, Joanna Warren, Simon C. Barry, Shane T. Grey

**Affiliations:** 1 Transplantation Immunology Laboratory, Garvan Institute of Medical Research, Darlinghurst, NSW Australia; 2 Translation Science Pillar, Garvan Institute of Medical Research, Darlinghurst, NSW Australia; 3 Department of Immunology, Harvard Medical School, Boston, MA USA; 4 Evergrande Center for Immunologic Diseases, Harvard Medical School and Brigham and Women’s Hospital, Boston, MA USA; 5 Robinson Research Institute, Adelaide Medical School, University of Adelaide, Adelaide, SA Australia; 6 School of Biotechnology and Biomolecular Sciences, Faculty of Science, University of New South Wales, Sydney, NSW Australia

**Keywords:** Beta cells, Diabetes, Enhancer hubs, Glucocorticoids, Islets, Metabolism, NEMO, NF-κB, p65, *TNFAIP3*

## Abstract

**Aims/hypothesis:**

NF-κB activation unites metabolic and inflammatory responses in many diseases yet less is known about the role that NF-κB plays in normal metabolism. In this study we investigated how *RELA* impacts the beta cell transcriptional landscape and provides network control over glucoregulation.

**Methods:**

We generated novel mouse lines harbouring beta cell-specific deletion of either the *Rela* gene, encoding the canonical NF-κB transcription factor p65 (βp65KO mice), or the *Ikbkg* gene, encoding the NF-κB essential modulator NEMO (βNEMOKO mice), as well as βA20Tg mice that carry beta cell-specific and forced transgenic expression of the NF-κB-negative regulator gene *Tnfaip3*, which encodes the A20 protein. Mouse studies were complemented by bioinformatics analysis of human islet chromatin accessibility (assay for transposase-accessible chromatin with sequencing [ATAC-seq]), promoter capture Hi-C (pcHi-C) and p65 binding (chromatin immunoprecipitation–sequencing [ChIP-seq]) data to investigate genome-wide control of the human beta cell metabolic programme.

**Results:**

*Rela* deficiency resulted in complete loss of stimulus-dependent inflammatory gene upregulation, consistent with its known role in governing inflammation. However, *Rela* deletion also rendered mice glucose intolerant because of functional loss of insulin secretion. Glucose intolerance was intrinsic to beta cells as βp65KO islets failed to secrete insulin ex vivo in response to a glucose challenge and were unable to restore metabolic control when transplanted into secondary chemical-induced hyperglycaemic recipients. Maintenance of glucose tolerance required *Rela* but was independent of classical NF-κB inflammatory cascades, as blocking NF-κB signalling in vivo by beta cell knockout of *Ikbkg* (NEMO), or beta cell overexpression of *Tnfaip3* (A20), did not cause severe glucose intolerance. Thus, basal p65 activity has an essential and islet-intrinsic role in maintaining normal glucose homeostasis. Genome-wide bioinformatic mapping revealed the presence of p65 binding sites in the promoter regions of specific metabolic genes and in the majority of islet enhancer hubs (~70% of ~1300 hubs), which are responsible for shaping beta cell type-specific gene expression programmes. Indeed, the islet-specific metabolic genes *Slc2a2*, *Capn9* and *Pfkm* identified within the large network of islet enhancer hub genes showed dysregulated expression in βp65KO islets.

**Conclusions/interpretation:**

These data demonstrate an unappreciated role for *RELA* as a regulator of islet-specific transcriptional programmes necessary for the maintenance of healthy glucose metabolism. These findings have clinical implications for the use of anti-inflammatories, which influence NF-κB activation and are associated with diabetes.

**Graphical Abstract:**

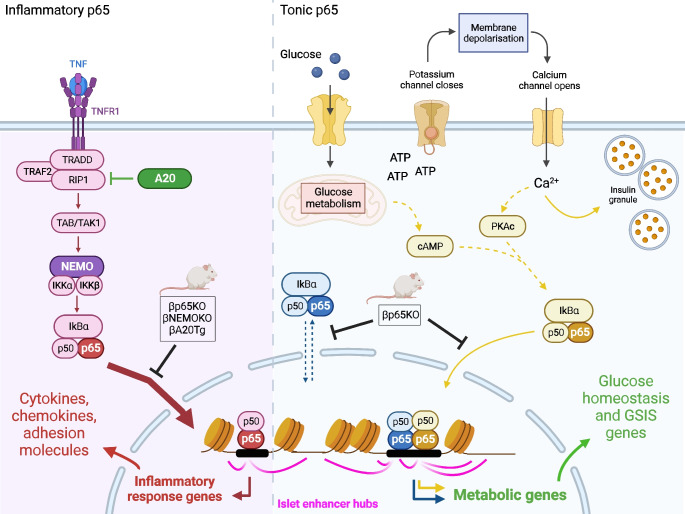

**Supplementary Information:**

The online version of this article (10.1007/s00125-023-05931-6) contains peer-reviewed but unedited supplementary material.


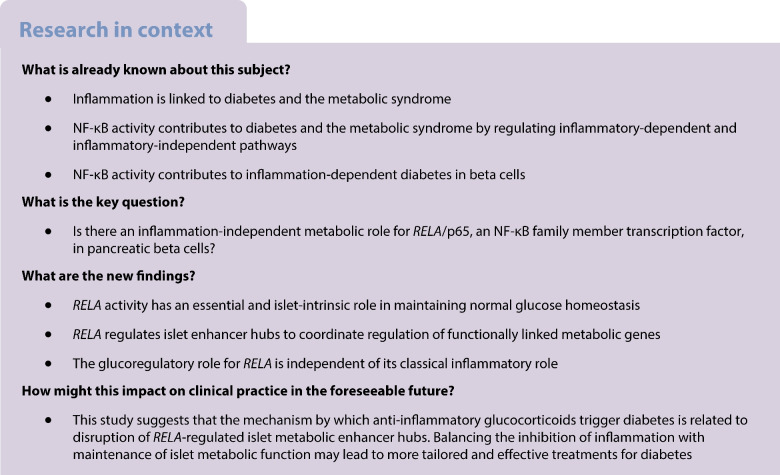


## Introduction

Regulation of correct blood glucose levels is essential to fuel the metabolic demands of the body and is therefore essential for life. A normal physiological glucose range is maintained through the coordinated efforts of glucose-sensing and glucose-responding tissues, including the brain, the liver, muscle and the pancreatic islets of Langerhans [1]. Disruptions to this tightly orchestrated glucoregulatory system can lead to hyperglycaemia and the metabolic disorder of diabetes, which include decreased glucose usage, increased glucose production, a failure to secrete sufficient quantities of insulin, and insulin resistance [2].

The NF-κB transcription factor family comprises DNA-binding components activated by canonical signalling, such as those encoded by *RELA* (p65), *REL* (c-Rel) and *NFKB1* (p105), as well as transcription factors activated by non-canonical signalling, such as those encoded by *RELB* (RelB) and *NFKB2* (p100) [3]. Classically assigned to an inflammatory role, aberrant canonical NF-κB activity and non-canonical NF-κB activity both contribute to insulin resistance and diabetes [4]. Decreased expression of IκB kinase (IKK)-β, which activates canonical NF-κB, or administration of salicylates, which inhibit IKK-β, protects from insulin resistance [5]. In contrast, IKK-β activation in hepatocytes drives insulin resistance in the liver in response to ageing or a high fat diet [6], and myeloid-intrinsic IKK-β activation drives peripheral insulin resistance in muscle and fat tissue [7]. Further, specific deletion of the p65 transcription factor, encoded by *Rela*, in hepatocytes [8] but not adipose tissue [9] improves liver insulin sensitivity, highlighting the potential for tissue-specific effects of canonical NF-κB activity in metabolic dysfunction. In addition to canonical-mediated metabolic dysfunction, activation of the non-canonical NF-κB pathway can also promote dysregulated glucose metabolism. Accumulation of the non-canonical NF-κB-inducing kinase (NIK) in obesity drives insulin resistance in the liver [10] and muscle [11] and impairs insulin secretion in pancreatic beta cells [12], whereas deletion of *Map3k14*, which encodes NIK, renders mice hypoglycaemic and improves glucose tolerance [10]. Collectively, these findings link canonical and non-canonical NF-κB activity to diabetes and the metabolic syndrome. Further to this, deleterious islet factors including inflammatory cytokines [13, 14], hyperglycaemia [15], amyloid deposition [16] and transplant reactions [17] can activate NF-κB in both autoimmune type 1 diabetes and type 2 diabetes and contribute to beta cell damage and functional failure. In the context of healthy metabolism, forced expression of the mutant form of nuclear factor of kappa light polypeptide gene enhancer in B cells inhibitor, alpha (IκBα) in beta cells to inhibit p65 was associated with a severe loss of glucose tolerance characterised by dampened insulin secretion [18]. These data link NF-κB activity to metabolic control in health and pathology. In this study we used murine models to investigate how *Rela* impacts the beta cell transcriptional landscape and provides network control over glucoregulation.

## Methods

### Animal studies

Animal studies using 6- to 12-week-old male and female mice housed under specific pathogen-free (SPF) conditions were approved by the Garvan Institute Animal Ethics Committee (no. 17_24). C57BL/6 mice were purchased from the Animal BioResource Centre (Sydney, Australia) and βA20Tg knock-in mice were generated at Ozgene (Australia) using murine *Tnfaip3* (cloned from cytokine-stimulated mouse islets; electronic supplementary material [ESM] Tables 1 and 2). Floxed C57BL/6 *Rela*/p65^loxP/loxP^ [19] and *Ikbkg*/NEMO^loxP/loxP^ [20] mice were a kind gift of M. Pasparakis (CECAD Research Center Cologne, Germany). Beta cell-specific deletion or expression was achieved by back crossing each line onto RIP-cre mice (Tg[Ins2-cre]25Mgn/J, C57BL/6 background; The Jackson Laboratory, USA, https://www.jax.org/strain/003573) [12].

### Minimal mass islet transplantation

In brief, and as described previously [13], 80 hand-picked isolated islets were transplanted under the kidney capsule of syngenic C57BL/6 mice with streptozotocin-induced hyperglycaemia. Diabetes was defined as a blood glucose level ≥16 mmol/l on 2 consecutive days following i.v. injection of alloxan (110 mg/kg). Blood glucose levels of non-fasted mice were determined using a FreeStyle Lite glucometer and blood glucose test strips (Abbott Diabetes Care) via tail tipping.

### Metabolic studies

In brief, and as described previously [12], i.p. GTTs were conducted following an overnight fast (16 h) and i.p. injection of dextrose at 2 g/kg (20% solution wt/vol) (Sigma-Aldrich). For i.v. GTTs, 1 g/kg glucose was administered intravenously. Glucose-stimulated insulin secretion (GSIS) assays were performed for islets ex vivo as described previously [12]. Blood glucose levels were assessed as described in the previous section and insulin levels were determined by specific ELISA (Cayman Chemical) [12].

### Immunohistochemistry and beta cell area determination

In brief, and as described previously [12], parallel pancreatic tissue sections were stained using standard protocols, buffers and diluents for insulin (rabbit anti-mouse insulin polyclonal antibody; 4590, Cell Signaling Technology), followed by incubation with horseradish peroxidase (HRP)-labelled polymer-conjugated goat anti-rabbit IgG (Dako EnVision+ System) and counterstaining with haematoxylin. Beta cell area was quantified from the total area (insulin-positive cells compared with non-positive tissue) using ImageJ (v1.53i; https://imagej.nih.gov) on consecutive pancreatic serial sections cut at 200 µm intervals. Beta cell mass (per mg) was calculated by multiplying the relative insulin-positive area by the mass of the isolated pancreas before fixation. Images were captured using a Leica DM 4000 or Leica DM 6000 Power Mosaic microscope (Leica Microsystems).

### Immunoblot analysis

Membranes were incubated using standard techniques, diluents and buffers with anti-A20 (56305/D13H3), anti-IκBα (9242), anti-phospho-IκBα (2859/I4D4), anti-IKKγ (2585), anti-JNK (9252), anti-phospho-JNK (9255), anti-p65 (6956/L8F6) all sourced from Cell Signaling Technology, anti-mCherry (ab183628) (Abcam) or anti-β-actin (AC-15) (Sigma-Aldrich) antibodies, followed by labelling with the HRP-conjugated secondary antibody goat-anti-mouse IgG Fc (Pierce Antibodies) or donkey-anti-rabbit IgG (GE Life Sciences). HRP conjugates were visualised using an ECL detection kit (GE Life Sciences).

### Real-time quantitative PCR

Total RNA was extracted using the RNeasy Plus Mini Kit (Qiagen) and reverse transcribed using the Quantitect Reverse Transcription Kit (Qiagen). Primers were designed using sequences from GenBank and synthesised by Sigma-Aldrich (ESM Table 3) or Taqman probes (Thermo Fisher Scientific) were used (Mm00446229_m1; Mm00499260_m1; Mm00510343_m1; Mm03024075_m1). PCR reactions were performed on the LightCycler 480 Real Time PCR System (Roche) using the PowerUP SYBR Green Master Mix or TaqMan Gene Expression MasterMix (Applied Biosystems). *PPIA* (also known as *CPH2*) and *ACTB* were used as housekeeping genes and data were analysed using the 2^–ΔΔCt^ method. Initial denaturation was performed at 95°C for 10 s; this was followed by a three-step cycle consisting of 95°C for 15 s (4.8°C/s, denaturation), 63°C for 30 s (2.5°C/s, annealing) and 72°C for 30 s (4.8°C/s, elongation). A melting curve analysis was performed after completion of 45 cycles using the following conditions: 95°C for 2 min, 40°C for 3 min and a gradual increase to 95°C with 25 acquisitions/°C.

### Bioinformatic analysis of islet enhancer hubs

Human islet gene networks were investigated using the assay for transposase-accessible chromatin with sequencing (ATAC-seq) based on genomic datasets including eight donors without diabetes generated by Bysani et al [21] downloaded from the GEO repository (accession no. GSE129383). Promoter capture Hi-C (pcHi-C) data and the corresponding enhancer hubs were obtained from Miguel-Escalada, et al [22]. High-confidence islet pcHi-C interactions (Capture HiC Analysis of Genomic Organisation [CHiCAGO] score >5) were loaded into the WashU Epigenome Browser along with RELA chromatin immunoprecipitation–sequencing (ChIP-seq; coverage peaks) data obtained from the Encyclopedia of DNA Elements (ENCODE Consortium) repository [23], ATAC-seq profiles and islet chromatin states from the NIH Roadmap Epigenomics Mapping Consortium, and H3K4me3 (trimethylation of histone H3 at lysine 4) and H3K4me1 (methylation of histone H3 at lysine 4) ChIP-seq signals from the International Human Epigenome Consortium (IHEC).

### ATAC-seq data processing

The libraries were trimmed to remove Nextera adapters using cutadapt (https://github.com/marcelm/cutadapt; TU Dortmund University). Trimmed reads were aligned to the GRCh37 genome using Bowtie2 (https://github.com/BenLangmead/bowtie2; Johns Hopkins University) with an ‘-X 2000’ setting. Quality trimming was performed with option ‘*-q 10*’ and ‘*-F 2828*’ using Samtools (https://github.com/samtools/samtools). Duplicate reads were removed using Picard (https://broadinstitute.github.io/picard/command-line-overview.html; Broad Institute). Mitochondrial reads, reads mapping to regions with an anomalous and unstructured signal (ENCODE hg19) and high signal regions on the nuclear genome that show sequence homology with the mitochondrial genome were excluded using BEDTools (https://bedtools.readthedocs.io/en/latest/; University of Utah). For peak calling the read start sites were adjusted to represent the centre of the Tn5 transposase binding event. Peaks were called from ATAC-seq data using MACS2 (https://pypi.org/project/MACS2/; Dana-Farber Cancer Institute), and HINT-ATAC (https://github.com/CostaLab/reg-gen; RWTH Aachen University) was used to identify footprints within the ATAC-seq peaks called from merged reads extracted from nucleosomal-free regions (Nfr) and regions bound by one nucleosome (1N) with the parameters ‘*--atac-seq --paired-end --organism*=*hg19*’. Intersection of genomic regions was performed using BEDTools. Enrichment of Gene Ontology (GO) terms and gene sets was performed using the ChIP-Enrich tool (http://chip-enrich.med.umich.edu/; University of Michigan).

### Statistics

All data are presented as means ± SEM or ± SD. Two-way Student’s *t* tests were performed to determine the statistical difference between groups. A *p* value <0.05 was considered significant. For transplant studies, data were plotted as blood glucose over time and analysed as AUCs. Tests were conducted using Prism (v8) software (GraphPad Software, USA).

## Results

### Characterisation of islets from beta cell-specific ***Rela***-deficient mice

**Fig. 1:**
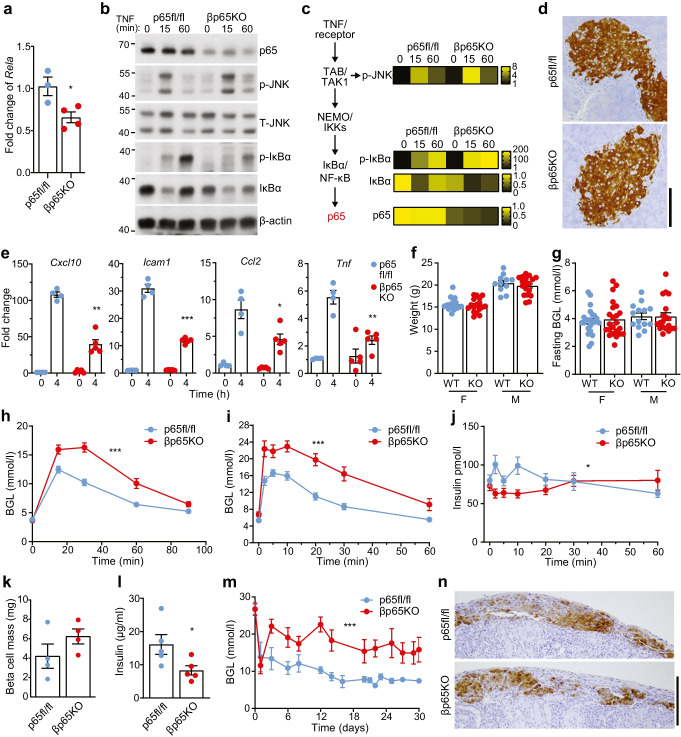
Beta cell-specific p65 knockout dampens islet inflammation but impairs insulin secretion in response to a glucose challenge. (**a**) Real-time quantitative PCR analysis of *Rela* mRNA in islets isolated from littermate mice wild-type for p65 (p65fl/fl) or with beta cell-specific knockout of p65 (βp65KO). (**b**) Immunoblot of lysates from islets isolated from p65fl/fl or βp65KO littermate mice and stimulated with recombinant TNF for the times indicated. Proteins (kDa) assessed included components of the canonical NF-κB signalling pathway, phosphorylated and total JNK (p-JNK and T-JNK, respectively) and a β-actin loading control. Representative of three independent experiments. (**c**) Cumulative densitometry (relative units) of immunoblots represented in (**b**), illustrated as heat maps. Data compared against wild-type floxed (fl) 0 h sample in each blot. The flow diagram of major signalling nodes illustrates the position of each signalling event with respect to the transcription factor *Rela*/p65 (red). (**d**) Insulin-stained pancreatic sections (scale bar: 100 µm) from 8 week old female mice of the indicated genotypes. (**e**) Real-time quantitative PCR analysis of inflammatory mRNAs in islets isolated from littermate p65fl/fl or βp65KO mice and stimulated with TNF for the times indicated. Data are fold change relative to no TNF stimulation. (**f**) Weight and (**g**) fasting blood glucose levels of mice with or without beta cell-specific knockout of p65. F, female; M, male. (**h**, **i**) Blood glucose levels were monitored following an (**h**) i.p. GTT (2 g/kg glucose) (p65fl/fl, *n*=17; βp65KO, *n*=17) or (**i**) i.v. GTT (1 g/kg glucose) (p65fl/fl, *n*=9; βp65KO, *n*=9) in female mice. (**j**) Blood insulin levels (pmol/l) were measured following i.v. injection in (**i**) (p65fl/fl, *n*=9; βp65KO, *n*=9). (**k**) Beta cell mass and (**l**) in vitro GSIS assay (20 mmol/l) in islets isolated from mice with or without beta cell-specific knockout of p65. (**m**) Blood glucose levels before (day 0) and following minimal mass transplant of islets from mice with or without beta cell-specific knockout of p65 under the kidney capsule of wild-type syngeneic diabetic recipients (βp65KO, *n*=7; p65fl/fl, *n*=4). (**n**) Insulin-stained sections of islet graft 30 days post transplant (scale bar: 100 µm). Statistical analysis was performed using Student’s *t* tests (**a**, **e–g**, **k**, **l**) or AUCs (**h–j**, **m**). Data are means ± SEM. *p<0.05, **p<0.01, ***p<0.001. BGL, blood glucose level; TAB, TAK1-binding protein; TAK1, TGF-β-activated kinase 1

We generated βp65KO mice that harbour beta cell-specific knockout of the *Rela* gene, which encodes the p65 protein. Islets isolated from βp65KO mice showed reduced levels of *Rela* mRNA and a >90% reduction in p65 protein (Fig. 1a–c), with residual *Rela* and p65 levels most likely due to expression in non-beta cells [24] (Fig. 1d). Stimulation of βp65KO islets with human recombinant TNF, which activates both NF-κB and the MAPK family member Jun N-terminal kinase (JNK) downstream of tumour necrosis factor receptor 1 (TNFR1) [25], triggered normal activation (e.g. phosphorylation and degradation kinetics, respectively) of IκBα and JNK, similar to that observed for control islets (Fig. 1b, c). However, βp65KO islets showed a reduced inflammatory stimulus response to TNF, exemplified by dampened induction of the islet-expressed inflammatory factors [14, 26] *Cxcl10*, *Icam1*, *Ccl2*, *Tnf*, *Cxcl1* and *Tnfaip3* (Fig. 1e; ESM Fig. 1a). In addition, IκBα expression is under the transcriptional control of NF-κB and βp65KO islets exhibited a ≥50% reduction in steady-state levels of IκBα protein (Fig. 1b, c). Therefore, deletion of *Rela* in beta cells did not perturb proximal TNF-activated signalling pathways, but limited the stimulus-induced expression of NF-κB-regulated inflammatory genes.

### ***Rela* regulates glucose tolerance at the level of the beta cell**

Female βp65KO mice showed both normal weight (Fig. 1f) and random blood glucose levels (Fig. 1g) at 8 weeks of age, but surprisingly exhibited profound glucose intolerance (i.p. GTT) compared with wild-type p65fl/fl littermates (Fig. 1h, ESM Fig 3a); data for male are shown in ESM Fig 2a. It should be noted that non-floxed wild-type and heterozygous Cre mice exhibited normal glucose tolerance (ESM Fig. 4). These results were subsequently extended with i.v. GTT studies in female mice (Fig. 1i, ESM Fig. 3b) and male mice (ESM Fig 2b). Glucose intolerance in βp65KO mice was due to a severely blunted glucose-stimulated insulin secretory response (Fig. 1j). Morphometric analysis showed that βp65KO mice exhibited a similar beta cell mass to p65fl/fl mice, eliminating a difference in endocrine mass as the driver of glucose intolerance in βp65KO mice (Fig. 1d, k). The glucose-stimulated insulin secretory defect was intrinsic to islets, as islets isolated from βp65KO mice showed defective GSIS (Fig. 1l) and, when transplanted under the kidney capsule of diabetic wild-type syngeneic recipients, islets from βp65KO mice failed to restore normal euglycaemia post transplant (Fig. 1m, n) without showing a change in beta cell mass (data not shown). Therefore, βp65KO islets show an islet-intrinsic insulin secretion defect.

### Impact of beta cell-specific deletion of NEMO on NF-κB and glucose intolerance

**Fig. 2:**
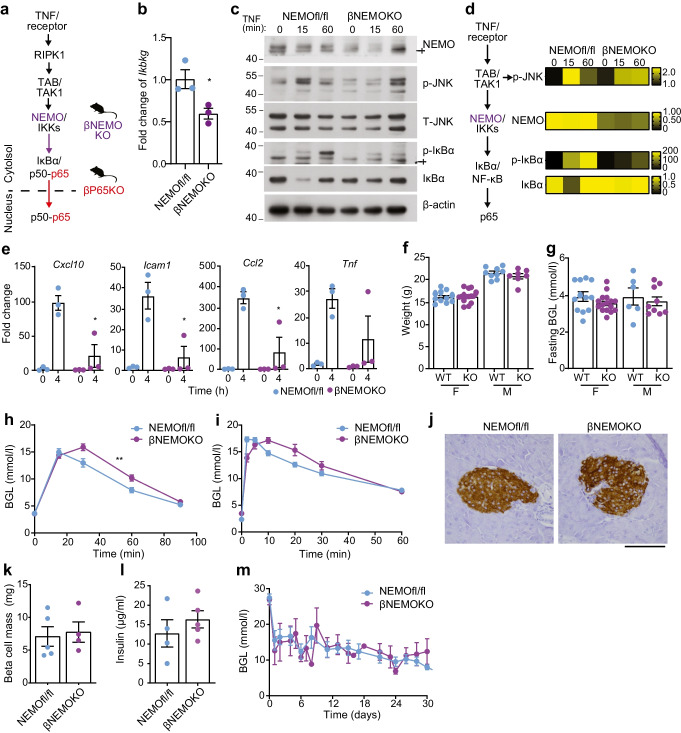
Beta cell-specific knockout of NEMO blunts the islet inflammatory response with subtle impairment of glucose tolerance. (**a**) Schematic illustration of the major signalling nodes in canonical NF-κB signalling and mouse models used in this study. βp65KO, red; βNEMOKO, purple. (**b**) Real-time quantitative PCR analysis of *Ikbkg* mRNA levels in islets isolated from mice with NEMO (NEMOfl/fl) or with beta cell-specific knockout of NEMO (βNEMOKO). (**c**) Immunoblot of lysates from islets isolated from NEMOfl/fl or βNEMOKO mice and stimulated with TNF for the times indicated. Proteins (kDa) assessed included components of the canonical NF-κB signalling pathway and a β-actin loading control. Representative of three independent experiments. † = non-specific band. (**d**) Cumulative densitometry (relative units) of immunoblots represented in (**c**), illustrated as heat maps. Data compared against the NEMOfl/fl 0 h sample in each blot. Flow diagram of major signalling nodes illustrates the position of each signalling event with respect to NEMO (purple). (**e**) Real-time quantitative PCR analysis of inflammatory mRNAs from islets isolated from littermate NEMOfl/fl or βNEMOKO mice and stimulated with TNF for the times indicated. Data are fold change relative to no TNF stimulation. (**f**) Weight and (**g**) fasting blood glucose levels of 8 week old mice with or without beta cell-specific knockout of NEMO. F, female; M, male. (**h**, **i**) Blood glucose levels were monitored following an (**h**) i.p. GTT (2 g/kg glucose) (NEMOfl/fl, *n*=9; βNEMOKO, *n*=15) or (**i**) i.v. GTT (1 g/kg glucose) (NEMOfl/fl, *n*=3; βNEMOKO, *n*=5) in 8 week old female mice. (**j**) Insulin-stained pancreatic sections (scale bar:100 µm) from 8 week old female mice of the indicated genotypes. (**k**) Beta cell mass and (**l**) in vitro GSIS assay (20 mmol/l) in islets from mice with or without beta cell-specific KO of NEMO. (**m**) Blood glucose levels before (day 0) and following minimal mass transplant of islets from mice with beta cell-specific knockout of NEMO (*n*=6) or from NEMOfl/fl control mice (*n*=10) into wild-type syngeneic diabetic recipients. Statistical analysis was performed Student’s *t* test (**b**, **e–g**, **k**, **l**) or AUCs (**h**, **i**, **m**). Data are means ± SEM. *p<0.05, **p<0.01. BGL, blood glucose level; RIPK1, receptor-interacting serine/threonine-protein kinase 1; TAB, TAK1-binding protein; TAK1, TGF-β-activated kinase 1

To test if the loss of insulin secretory response in βp65KO mice was a generalisable consequence of NF-κB inhibition or was dependent on inflammatory NF-κB activation, we generated βNEMOKO mice that harbour beta cell-specific deletion of the *Ikbkg* gene. *Ikbkg* encodes the NF-κB essential modulator (NEMO) subunit of the activating IκB kinase (IKK) protein complex [3] and is required for IKK-β-mediated phosphorylation and degradation of IκBα and subsequent activation of p65 (Fig. 2a). Islets from βNEMOKO mice showed reduced *Ikbkg* mRNA and NEMO protein levels (Fig. 2b, c). Following TNF stimulation, βNEMOKO islets exhibited markedly reduced phosphorylation and degradation of IκBα, with no impact on JNK pathway activation (Fig. 2c, d). TNF-stimulated βNEMOKO islets also exhibited a reduction in the upregulation of NF-κB-regulated inflammatory genes (Fig. 2e; ESM Fig. 1b), showing that NEMO is essential for TNF-induced inflammatory responses in beta cells. Female βNEMOKO mice showed both normal weight (Fig. 2f) and random blood glucose levels (Fig. 2g) at 8 weeks of age. However, different from βp65KO mice, βNEMOKO mice exhibited a subtle change in glucose intolerance that was less pronounced in female mice and chiefly observed in the i.p. GTT (Fig. 2h, ESM Fig. 3c) and not the i.v. GTT (Fig. 2i, ESM Fig. 3d). Extended analysis revealed a potential sex difference in the i.p. GTT response in male βNEMOKO mice (ESM Fig. 2c), which was not replicated in the i.v. GTT response (ESM Fig. 2d). In addition, different from βp65KO mice, βNEMOKO mice exhibited normal beta cell insulin staining and beta cell mass (Fig. 2j, k) and normal GSIS (Fig. 2l) and islets from βNEMOKO mice were able to restore euglycaemia in diabetic transplant recipients (Fig. 2m).

### Impact of beta cell-specific transgenic overexpression of ***Tnfaip3*** on NF-κB and glucose intolerance

**Fig. 3:**
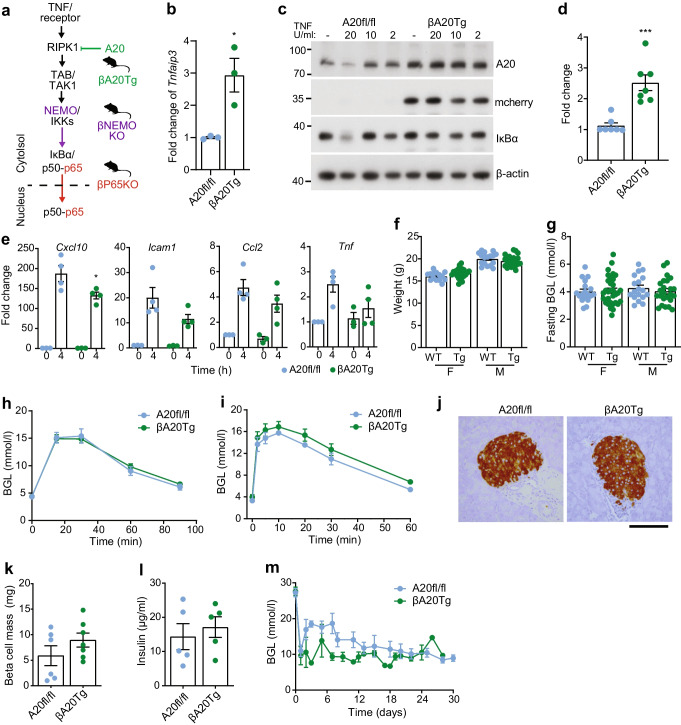
Beta cell-specific overexpression of A20 blunts the islet inflammatory response without impairing glucose tolerance. (**a**) Schematic illustration of the major signalling nodes in canonical NF-κB signalling and mouse models used in this study. (**b**) Real-time quantitative PCR analysis of *Tnfaip3* mRNA in islets isolated from mice with (βA20Tg) or without (A20fl/fl) beta cell-specific overexpression of A20. (**c**) Immunoblot of lysates from islets isolated from A20fl/fl or βA20Tg mice stimulated for 15 min with the indicated doses of TNF. Proteins (kDa) assessed were A20 and mCherry, a marker of transgene expression, and the NF-κB inhibitor IκBα. β-actin was probed as the loading control. Representative of three independent experiments. (**d**) Cumulative densitometry (relative units) of A20 protein levels. (**e**) Real-time quantitative PCR analysis of inflammatory mRNAs in islets isolated from A20fl/fl or βA20Tg mice and stimulated with TNF for the times indicated. Data are fold change relative to no TNF stimulation. (**f**) Weight and (**g**) fasting blood glucose levels of 8 week old mice with or without beta cell-specific overexpression of A20. F, female; M, male. (**h**, **i**) Blood glucose levels were monitored following an (**h**) i.p. GTT (2 g/kg glucose) (A20fl/fl, *n*=7; βA20Tg, *n*=13) and (**i**) i.v. GTT (1 g/kg glucose) (A20fl/fl, *n*=4; βA20Tg, *n*=7) in 8 week old female mice. (**j**) Insulin-stained pancreatic sections (scale bar: 100 µm) from 8 week old female mice of indicated genotypes. (**k**) Beta cell mass and (**l**) in vitro GSIS assay (20 mmol/l) in islets isolated from mice with or without beta cell-specific overexpression of A20. (**m**) Blood glucose levels before (day 0) and following minimal mass transplant of islets from mice with beta cell-specific overexpression of A20 (βA20Tg, *n*=3) or from fl/fl control mice (A20fl/fl, *n*=9) into wild-type syngeneic diabetic recipients. Statistical analysis was performed using Student’s *t* tests (**a**, **c**–**f**, **j**, **k**) or AUCs (**g**, **h**, **l**). Data are means ± SEM. *p<0.05, ***p<0.001. BGL, blood glucose level, ; RIPK1, receptor-interacting serine/threonine-protein kinase 1; TAB, TAK1-binding protein; TAK1, TGF-β-activated kinase 1

To further test if the loss of insulin secretory response in βp65KO mice was a generalisable consequence of NF-κB inhibition, we explored the metabolic impact of forcing *Tnfaip3* expression in beta cells by generating βA20Tg mice (ESM Fig. 5). *Tnfaip3* encodes the cytoplasmic ubiquitin-editing enzyme A20 [27], a master negative regulator of NF-κB signalling in islets [25, 28]. A20 inhibits NF-κB signalling upstream of NEMO and p65 (Fig. 3a) [25] and *Tnfaip3* expression is tightly regulated in islets by NF-κB signalling cascades to form a physiological NF-κB negative feedback loop [29]. Islets from βA20Tg mice showed two- to fourfold higher levels of *Tnfaip3* mRNA and A20 protein than A20fl/fl littermates (Fig. 3b–d). βA20Tg islets also showed a reduced TNF stimulatory response, with reduced IκBα degradation (Fig. 3c) and inflammatory gene expression (Fig. 3e; ESM Fig. 1c), mirroring the findings from ectopic *Tnfaip3* gene engineering [28]. Female βA20Tg mice exhibited normal weight gain (Fig. 3f) and random blood glucose levels (Fig. 3g) at 8 weeks of age. They also showed normal glucose tolerance following an i.p. GTT (Fig. 3h; ESM Fig. 3e) and i.v. GTT (Fig. 3i; ESM Fig. 3f), with normal beta cell architecture (Fig. 3j), beta cell mass (Fig. 3k) and insulin secretory function in vitro (Fig. 3l) and in vivo (Fig. 3m). Male βA20Tg mice showed a normal i.p. GTT response (ESM Fig. 2e) and mild glucose intolerance following an i.v. GTT (ESM Fig. 2f).

### p65 interacts with both proximal promoters and long-range enhancer hubs in islets

**Fig. 4:**
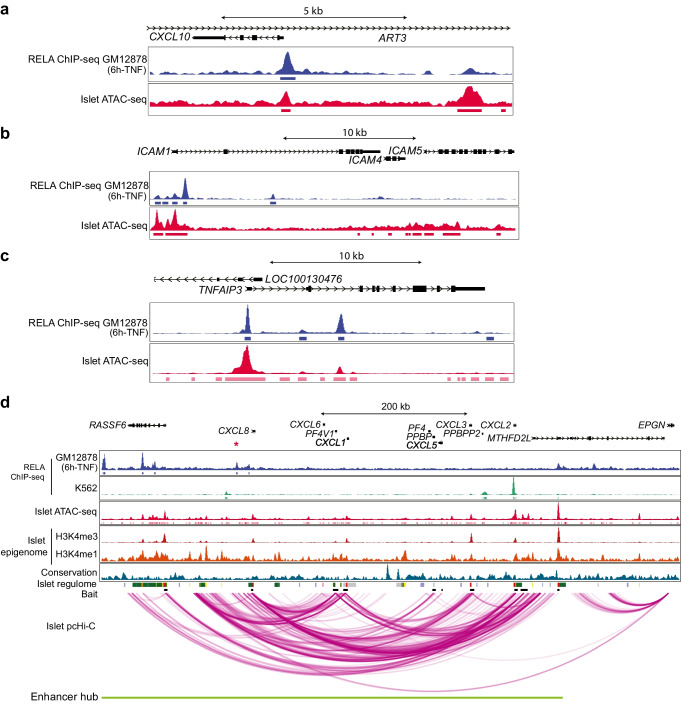
Islet-derived inflammatory genes harbour p65 binding sites. (**a–c**) Representative plots of p65 ChIP-seq and corresponding accessibility signal enrichment at the (**a**) *CXCL10*, (**b**) *ICAM1* and (**c**) *TNFAIP3* locuses. ChIP-seq and ATAC-seq peaks are shown underneath the respective signal coverage tracks. (**d**) p65 ChIP-seq enrichment, epigenomic annotations and high-confidence pcHi-C interactions from islet samples encompassing the *CXCL1* locus. p65 ChIP-seq data were obtained from ENCODE. ATAC-seq data were obtained from Bysani et al [21]. All browser views were generated using the WashU Epigenome Browser. Only the significant pcHi-C loops within the broadcast region are shown. ChIP, chromatin immunoprecipitation

Severely disrupted glucose tolerance and the loss of the insulin secretory response in βp65KO mice was not a generalisable consequence of NF-κB inhibition but is specific to *Rela* deficiency. *Rela* is therefore required to maintain beta cell glucose responsiveness and metabolic control independent of *Rela*’s classical role in mediating inflammatory signalling. As *RELA* encodes a transcription factor, and to investigate genome-wide control of the beta cell metabolic programme, we analysed human islet chromatin accessibility (ATAC-seq) and human p65 binding (ChIP-seq) datasets [21, 23]. As ChIP-seq data identify p65-bound chromatin sites, and ATAC-seq reveals islet-specific chromatin accessibility, the colocalisation of peaks would indicate sites of active regulation by p65 in human islets. As proof of concept to the approach we first examined evidence for p65 binding to experimentally validated [14, 26, 29] islet-expressed NF-κB-regulated genes. Evidence of p65 binding was found in inflammatory gene loci (*CXCL10*, *ICAM1* and *TNFAIP3*; Fig. 4a–c), consistent with the blunted mRNA levels of these genes in TNF-stimulated βp65KO islets (Fig. 1e; ESM Fig. 1a). Interestingly, ChIP-seq analysis did not identify p65 binding in close proximity to *CXCL1* or *TNF* loci (Fig. 4d, ESM Fig. 6, respectively), despite the fact that the mRNA levels of *Cxcl1* and *Tnf* were found to be blunted in βp65KO islets (Fig. 1e; ESM Fig. 1a) but also in islets with forced expression of *Tnfaip3* (Fig. 3e; ESM Fig. 1c) [17]. To investigate this observation further, we took advantage of pcHi-C analysis performed in human pancreatic islets [22] to explore the high-resolution map of long-range chromatin interactions between gene promoters and distant regulatory elements. Subsequent analysis including of connectivity (pcHi-C) and co-regulation (enhancer hub annotation) revealed that the *CXCL1* and *TNF* loci each reside in defined islet super-enhancer hubs that also harbour *RELA* binding sites. These findings indicate that the co-regulation of key genes by p65 is coordinated through both proximal promoter interactions and long-range enhancer hubs (Fig. 4d; ESM Fig. 6).

### p65 provides network control over genes governing islet metabolic function

**Fig. 5:**
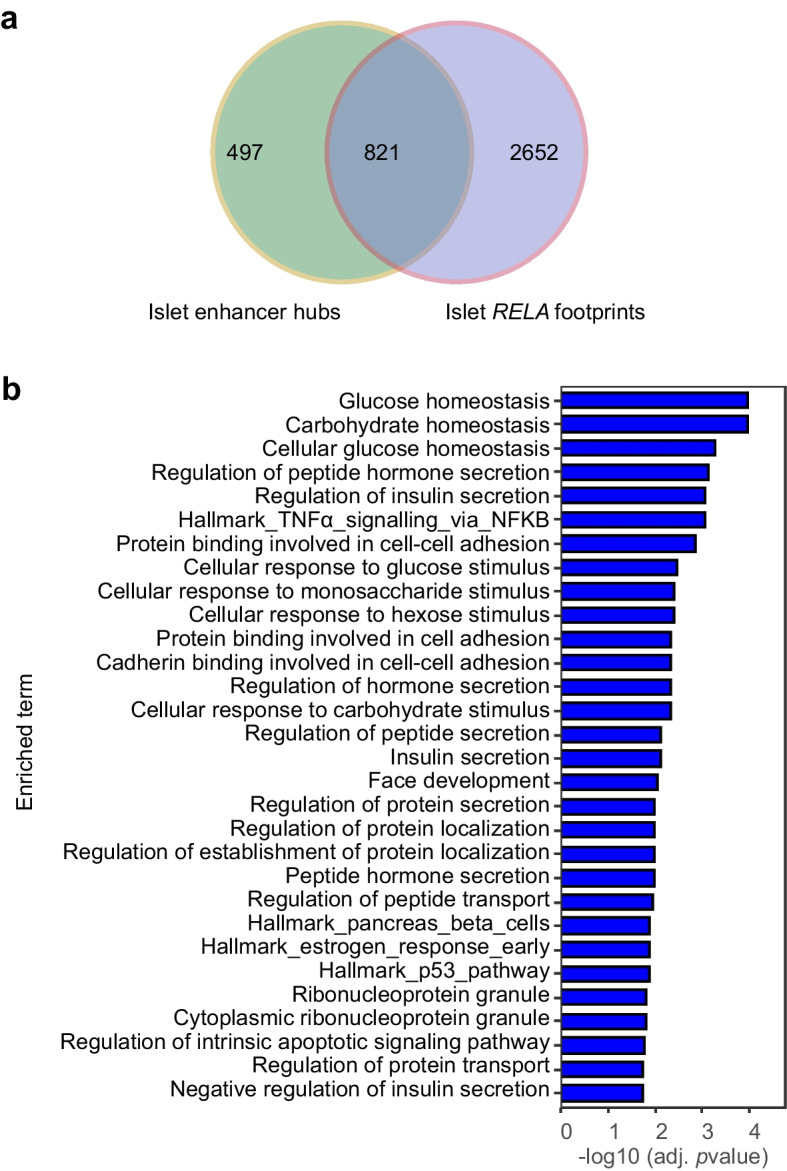
Human islets exhibit a large network of accessible p65 binding sites linked to genes governing metabolism. (**a**) Venn diagram showing the enrichment of islet-accessible p65 footprints in the islet enhancer hubs. p65 footprints were identified from islet ATAC-seq samples (*n*=8 healthy donors) available from Bysani et al [21] and intersected with islet enhancer hubs reported by Miguel-Escalada et al [22]. (**b**) Top 30 significantly enriched GO terms or gene sets for p65 footprints identified within the islet enhancer hubs. The footprint regions (*n*=1684 within the 821 enhancer hubs) were assigned to the nearest transcription start site to perform GO term ontology and gene set enrichment using the ChIP-Enrich tool [52]

To investigate the molecular networks driving dysregulated beta cell function we used *HINT-ATAC* and amalgamated p65 footprints identified from pooled ATAC-seq data generated from eight independent human islet preparations [21] and determined how many of the identified p65 footprints occur in islet enhancer hubs [22]. Consequently, we found that 62.3% of all (i.e. >1300 [22] islet enhancer hubs contained at least one p65 footprint (Fig. 5a). As human islet enhancer hubs have been described as encompassing genes important for islet metabolism, islet cell identity, differentiation and diabetes [22], these data underscore a potentially important role for p65 in these processes.

GO term analysis of all the islet-accessible p65 footprints located within the islet 3D enhancer hubs revealed the top 30 GO term hits to be enriched for metabolic and cell secretion gene sets (Fig. 5b). The top five hits were ‘glucose homeostasis’, ‘carbohydrate homeostasis’, ‘cellular glucose homeostasis’, ‘regulation of peptide hormone secretion’ and ‘regulation of insulin secretion’. ‘Negative regulation of insulin secretion’ and ‘hallmark_pancreas_beta_cells’ were also among the top 30 GO terms. Significantly, the GO gene set ‘hallmark_TNFα_signalling_via_NF-κB’ was among the top ten GO terms, supporting the interpretation that these newly identified metabolic gene hubs are regulated by p65.

### p65 provides network control over glucose sensing

**Fig. 6:**
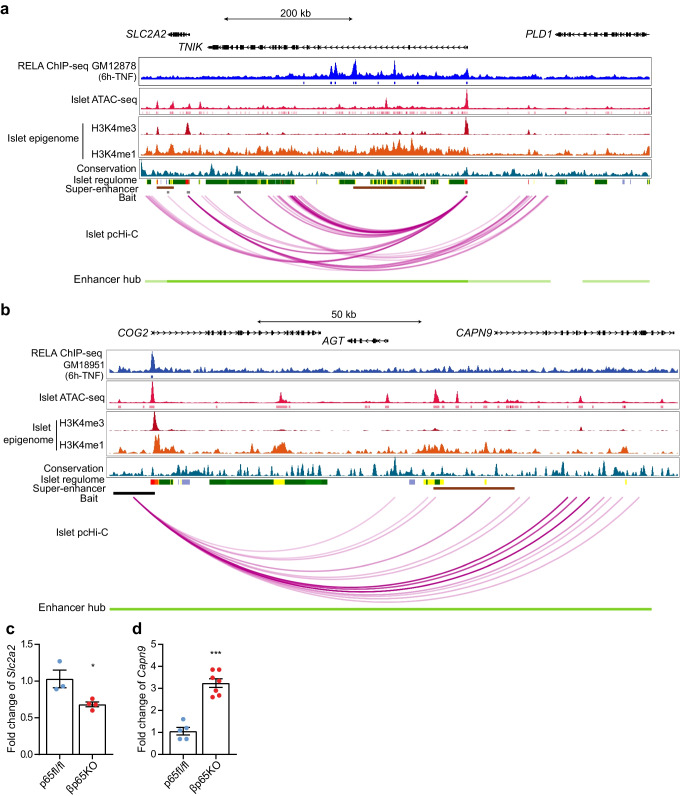
*SLC2A2* and *CAPN9* locuses form part of the islet enhancer hubs linked with p65 regulation. (**a**, **b**) p65 ChIP-seq enrichment, epigenomic annotations and high-confidence pcHi-C interactions from islet samples at the two candidate loci: (**a**) *SLC2A2* and (**b**) *CAPN9*. p65 ChIP-seq data were obtained from ENCODE. Islet pcHi-C interactions and super enhancers were obtained from Miguel-Escalada et al [22]. All browser views were generated using the WashU Epigenome Browser [53]. Only the significant pcHi-C loops within the broadcast region are shown. ChIP, chromatin immunoprecipitation. (**c**, **d**) Real-time PCR analysis of (**c**) *Slc2a2* and (**d**) *Capn9* in islets isolated from p65fl/fl and βp65KO mice. Statistical analysis was performed using Student’s *t* tests. Data are means ± SEM. *p<0.05, ***p<0.001

*SLC2A2* (which encodes glucose transporter 2 [GLUT2]) mediates the uptake of glucose into beta cells, is essential for GSIS and is associated with an increased risk for type 2 diabetes [30]. We found that the transcription start site of *SLC2A2* interacts strongly with the gene promoter of *TNIK* (which encodes TRAF2- and NCK-interacting kinase) in 3D space (Fig. 6a). The *TNIK* gene locus also contains p65 islet footprints and p65 ChIP-hits. *TNIK* has also been reported as a susceptibility locus associated with type 2 diabetes [23]. In addition, the *SLC2A2* gene promoter also forms part of an islet-selective enhancer hub (EHUB_918) that harbours chromatin interactions stemming from the *MIR569*, *TNIK*, *RNU1-70P* and *PLD1* promoters (Fig. 6a). We also identified the *CAPN9* gene, which contains type 2 diabetes SNPs [30] and belongs to the Calpain family of genes, which have been linked to type 2 diabetes when overexpressed by impairing insulin exocytosis in beta cells [31]. The *CAPN9* locus contains a super enhancer that interacts with the gene promoter of *COG2* (which encodes component of oligomeric Golgi complex 2) in 3D space (Fig. 6b). We conducted real-time quantitative PCR on islets isolated from βp65KO mice and their floxed wild-type littermates to test the effect of removing p65 on the levels of *Slc2a2* and *Capn9* under basal conditions. We subsequently found reduced levels of *Slc2a2* and increased levels of *Capn9* in βp65KO islets (Fig. 6c, d).

### ***RELA* interacts with islet enhancer hubs to provide network control over islet glycolysis genes**

These data demonstrate the presence of p65 binding sites in the majority of islet enhancer hubs that control metabolism, islet cell identity, islet differentiation and diabetes [22]. GO term analysis revealed p65 binding sites to be enriched in islet hubs that control metabolic genes, with glucose and carbohydrate homeostasis dominating the top three positions (Fig. 5b). Therefore, we next investigated p65 binding sites in genes that specifically govern glycolysis. We found that eight of 17 known glycolysis genes identified within the PANTHER Pathways database (http://www.pantherdb.org) (ESM Fig. 7) lie proximal to p65 binding sites (Table 1), with *ENO1* and *PKM* harbouring p65 binding sites within an islet super enhancer, and six glycolysis genes harbouring p65 binding sites within islet enhancer hubs (Table 1). Microarray analysis of steady-state βp65KO and wild-type littermates found that ten of 17 glycolysis genes with p65 binding sites at any location (proximal promoter, super enhancer, enhancer hub) show reduced expression (Table 1), with a significant reduction in the expression of *PFKL*, which encodes phosphofructokinase, an enzyme that has a rate-limiting effect on glycolysis [32], indicating that reduction in *PFKL* expression is likely to cause altered metabolism.

**Table 1:** Annotation of p65 binding sites in proximal promoters and enhancers of glycolytic genes, with impacts on gene expression

Glycolysis gene^a^	Position in pathway^b^	p65 binding site^c^	Islet accessible^d^	Islet super enhancers^e^	Islet enhancer hub^f^	KO vs WT logFC^g^	*p* value^h^
*HK1*	1	Yes	Yes	No	No	−0.2	0.07
*HK2*	1	Yes	Yes	No	No	−0.32	0.07
*HKDC1*	1	Yes	Yes	No	No	−0.15	0.77
*GPI*	2	No	Yes	No	Yes	−0.06	0.53
*PFKL*^i^	3	Yes	Yes	No	No	−0.16	0.05*
*PFKM*^i^	3	Yes	Yes	No	No	−0.15	0.2
*ALDOA*	4	Yes	Yes	No	No	−0.2	0.08
*TPI1*	5	No	Yes	No	Yes	0.05	0.71
*GAPDH*	6	No	Yes	No	Yes	–	–
*PGK1*	7	No	Yes	No	No	–	–
*PGAM1*	8	No	Yes	No	No	–	–
*PGAM2*	8	No	Yes	No	Yes	–	–
*PGAM4*	8	No	No	No	No	–	–
*ENO1*	9	Yes	Yes	Yes	No	–	–
*ENO2*	9	No	Yes	No	Yes	−0.16	0.12
*PKLR*	10	No	Yes	No	No	−0.09	0.75
*PKM*	10	Yes	Yes	Yes	Yes	−0.07	0.49

^a^Glycolysis genes were identified from the PANTHER Pathways database as indicated (ESM Fig. 7)

^b^Relative numbered position in the glycolytic pathway

Bioinformatic analysis of p65 interactome and chromatin features of glycolysis genes:

^c^Presence (yes) or absence (no) of p65 ChIP-seq peaks along the entire gene of interest. The p65 ChIP-seq peaks were derived from the GM12878 (TNF stim), GM18951 (TNF stim) and K562 cell lines as described in Miguel-Escalada et al [22]. The presence of ChIP-seq peak(s) from any of these cell lines at the gene of interest is reported as ‘yes’

^d^Presence (yes) or absence (no) of ATAC-seq peaks at the gene of interest. The ATAC-seq peaks were derived from islet samples as identified in Bysani et al [21]

^e^Presence (yes) or absence (no) of super enhancer(s) at the gene of interest. The super-enhancer annotations were derived from islet samples as identified in Miguel-Escalada et al [22]

^f^Presence (yes) or absence (no) of an enhancer hub at the gene of interest. The enhancer hub annotations were derived from the islet samples as identified in Miguel-Escalada et al [22]. A ‘no’ indicates that the gene is not a constituent of an islet hub [22].

^g^Microarray analysis of steady-state differential expression of each glycolytic gene in islets isolated from p65KO or wild-type littermates. Data are expressed as logFC expression

^h^*p* values (Student’s *t* test) for comparison in the previous column. *p≤0.05

^i^The rate-limiting steps in glycolysis

FC, fold change; KO, knockout; WT, wild-type

## Discussion

In this study we show that *RELA* shapes the beta cell transcriptional landscape by direct interaction with proximal promotors of metabolic target genes, as well as through remote enhancer hubs, providing coordinated control of beta cell-specific gene expression programmes responsible for glucose homeostasis. These data establish a fundamental role for basal levels of *RELA* in glucoregulation. These 3D islet enhancer hubs are enriched for DNA variants that impact the heritability of glucose homeostasis and insulin secretion [22], suggesting an important role for p65-dependent islet 3D hubs in islet function, but also describing how insulin secretion and glucoregulation may have been tuned evolutionarily.

The discovery of p65-dependent islet enhancer hubs that control glucoregulation increases our understanding of the impact of natural genetic variation in healthy metabolism. Indeed, common genetic polymorphisms (SNPs) can influence metabolic control [33], which raises the interesting possibility that naturally occurring polymorphisms within molecular components of the NF-κB signalling cascade, including *RELA*, could also contribute to human variance in glucoregulation. Indeed, genetic variance in the islet NF-κB regulator *TNFAIP3* [28] contributes to dysregulated beta cell inflammation and loss of islet metabolic function under stress conditions [17, 25], while *TNFAIP3* SNPs associate with diabetic complications [34]. Further, *TNFAIP3* exhibits complex crosstalk with the non-canonical NF-κB pathway, including a regulatory role in NIK activation in islets [25]. NIK activation is a mechanism of beta cell metabolic dysfunction in obesity through alteration of beta cell transcriptional programmes to favour reduced insulin output [12], but is not required for the development of diet-induced diabetes per se [35], suggesting that NIK activation is a cofactor in metabolic dysfunction. NIK is regulated by TNF receptor-associated factor (TRAF)2 and TRAF3 in beta cells [12] and, of interest, TRAF3 SNPs associate with type 2 diabetes risk and body weight variables in genome-wide association studies (data mined from the National Human Genome Research Institute–European Bioinformatics Institute [NHGRI-EBI] GWAS Catalog; summary statistics accession ID: GCST90132184; GCST010557; GCST009001; GCST008996). Further to this, the baculoviral inhibitors of apoptosis repeat-containing (BIRC)2 and BIRC3 proteins regulate TNF-induced TRAF2 and TRAF3 activity in beta cells [36], and beta cell deletion of BIRC2 and BIRC3 results in loss of metabolic function under inflammatory stress conditions [36]. These data highlight how investigating the influence of p65 on 3D networks of islet-specific metabolic genes through canonical and non-canonical NF-κB signalling pathways can enhance understanding of both healthy glucose homeostasis and metabolic dysfunction.

We identified GO terms for unexpected gene sets within the islet enhancer hubs, including ‘hallmark_estrogen_response_early’. Enrichment for oestrogen response genes matched with the more severe glucose intolerance observed in βp65KO female mice and, of interest, oestrogen has been reported to play a glucose-lowering role in humans and mice [37] by enhancing beta cell insulin secretion [38]. Further investigation into how NF-κB–oestrogen pathways intersect and influence sex-dependent insulin secretion and diabetes may have importance for addressing clinical inequalities in diabetes treatment [39]. More broadly, in fibroblasts NF-κB inhibition causes cellular reprogramming favouring aerobic glycolysis under basal conditions [40], while in cardiomyocytes NF-κB deletion protects against ischaemic reperfusion injury by preservation of calcium handling [41]. This is interesting as calcium flux is a critical determinant for insulin secretion in beta cells. These insights place *RELA* among a suite of factors that influence healthy metabolism, including external factors such as exercise [42], antibiotics and glucose-lowering drugs [43], as well as molecular components of glucose sensing and insulin release such as voltage-gated K^+^ channels [44].

This study has some limitations with regard to the use of animal strains and lines in the generation of beta cell-specific targeted gene deletions. Some Cre lines have been reported to exhibit glucose tolerance defects [45], and it has been shown that specific C57BL/6 lines that carry the *Nnt* mutation can display defects in glucose tolerance and insulin secretion [46]. To rule out these confounders we conducted rigorous control studies and found no glucose intolerance for the Cre recombinase-expressing mouse line nor for the gene-targeted floxed wild-type lines used in this study (ESM Fig. 4), mirroring reports from other studies using this Cre line [12]. The present results could be further validated by repeating these studies with p65 and NEMO lines, as well as the *Tnfaip3*/A20 transgenic lines, backcrossed onto C57BL/6N mice, and by using alternative Cre lines (e.g. Ins1Cre line).

The new concept that p65 plays a role in healthy metabolic homeostasis is intriguing in light of the central role that p65 specifically, and the NF-κB pathway in general, plays in regulating islet inflammatory homeostasis. Deletion or blocking of NF-κB/p65 activation in beta cells and islets prevents activation of islet-intrinsic inflammation [14, 47] and counters cell death pathways [28, 29, 48] through mechanisms that include a reduction in activity of islet-toxic nitric oxide pathways [28], but also has therapeutic potential to enhance islet survival in inflammatory transplant settings [13, 14, 17, 49]. Thus, our findings have clinical implications in that approaches to treating islet inflammation in diabetes by inhibiting NF-κB activation should consider potential beta cell toxic side effects. Indeed, these findings have a potentially broader application as anti-inflammatory glucocorticoids repress NF-κB-regulated genes by directly interrupting the interaction of p65 with the basal transcription machinery [50], and glucocorticoid use in patients can cause diabetes, in part because of direct effects on beta cell insulin synthesis and secretion [51]. This study suggests that one additional mechanism of action in glucocorticoid-induced diabetes may be glucocorticoid-dependent disruption of p65-dependent islet 3D enhancer hubs, thus disrupting the network control provided by p65 over islet metabolic transcriptional programmes. In contrast, unlike glucocorticoids, diabetes treatment with high-dose salicylates [5], anti-cytokine antibodies (TNF and IL1β) [2] or gene therapy approaches targeting NF-κB regulatory loops (e.g. A20) [13, 17] do not risk directly inhibiting basal NF-κB activity. By understanding the important role of p65-regulated islet 3D enhancer hubs in the coordinated regulation of functionally linked gene expression in islets and other metabolic tissues, it will be possible to develop more tailored therapies with improved patient outcomes.

## Supplementary Information

Below is the link to the electronic supplementary material.

Supplementary file1 (PDF 1568 KB)

## Data Availability

All data will be made available on request to the senior author.

## Abbreviations

ATAC-seq: Assay for transposase-accessible chromatin using sequencing
ChIP: Chromatin immunoprecipitation
ENCODE: Encyclopedia of DNA Elements
GO: Gene Ontology
GSIS: Glucose-stimulated insulin secretion
H3K4me3: Trimethylation of histone H3 at lysine 4
H3K4me1: Methylation of histone H3 at lysine 4
HRP: Horseradish peroxidase
IκBα: Nuclear factor of kappa light polypeptide gene enhancer in B cells inhibitor, alpha
IKK: IκB kinase
JNK: Jun N-terminal kinase
NEMO: NF-κB essential modulator
NIK: NF-κB-inducing kinase
pcHi-C: Promoter capture Hi-C
TRAF: TNF receptor-associated factor
