# Supplementary material for: RELA governs a network of islet-specific metabolic genes necessary for beta cell function
Source: Diabetologia. 2023 Jun 14;66(8):1516–31. doi: 10.1007/s00125-023-05931-6 (PMC10317895; doi:10.1007/s00125-023-05931-6)
Supplement: Supplementary file 1 — Supplementary file1 (PDF 1568 KB) [file 125_2023_5931_MOESM1_ESM.pdf]

`Electronic Supplementary Material

Electronic Supplementary Material Tables:

ESM Table 1 mA20 amplification primers.

**Coding sequence (CDS) Position**	**Sequence**	**Tm**
266-286	5’-CGAGGACCATGGCTGAACAAC-3’	56.7
3126-3107	5’-TGACCACAGATGTGCTCCGC-3’	56.7

ESM Table 2 Forward and Reverse primers for the insertion of restriction sites EcoRI and BamHI into mA20.

**Primer**	**Sequence**
mA20_reverse_EcoRI	5’-GCGCGAATTCGGACCTGTAATGTGTTCGCAC-3’
mA20_forward_BamHI	5’-AAATGGATCCGCCGCCACCATGGCTGAACAACTTCTTC CTC-3’

ESM Table 3

Mouse primers used for qRT-PCR analysis.

**Gene**	**Primer sequence**
Tnfaip3	F-5’-CCTGTCACCAACGCTCCAAG-3’<br>R-5’-ATTTCCAGTCCGGTGGCAAG-3’
Ccl2	F- 5'-GGTCCCTGTCATGCTTCTGG-3'<br>R- 5'-CCTGCTGCTGGTGATCCTCT-3'
Cph2	F- 5'-TGGACCAAACACAAACGGTTCC-3'<br>R- 5'-ACATTGCGAGCAGATGGGGT-3'
Cxcl1	F- 5'-TGGCTGGGATTCACCTCAAG-3'<br>R- 5'-TATGACTTCGGTTTGGGTGCAG-3'
Cxcl10	F- 5'-GACGGGCCAGTGAGAATGAG-3'<br>R- 5'-GTGTGTGCGTGGCTTCACTC-3'
Icam1	F- 5'-CCATGGGAATGTCACCAGGA-3'<br>R- 5'-ATCACGAGGCCCACAATGAC-3'
Tnf	F- 5'-ATGGCCCAGACCCTCACACT-3'<br>R- 5'-TGGTGGTTTGCTACGACGTG-3'

Electronic Supplementary Material Figures:

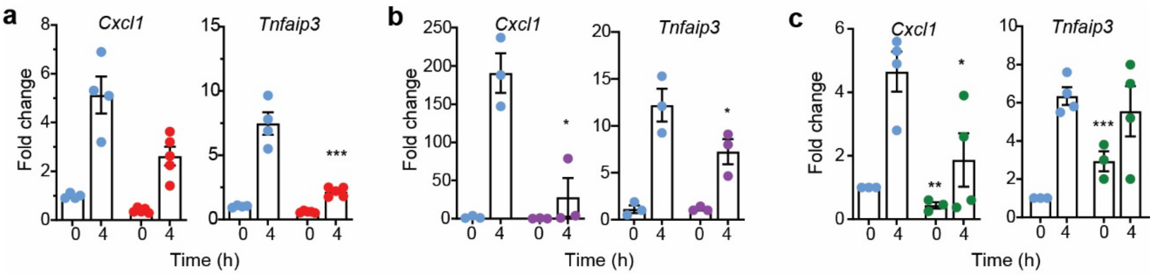

ESM Figure 1. Gene expression in gene modified islets. RTPCR analysis for *Cxcl1* and *Tnfaip3* from islets isolated from **(A)** βp65KO, **(B)** NEMOKO or **(C)** βA20Tg mice and their littler mate wild-type floxed controls, as indicated. Statistical analysis was performed Student's t-test, and data shown as the mean +/- s.e.m: *$P < 0.05$; ***$P < 0.001$.

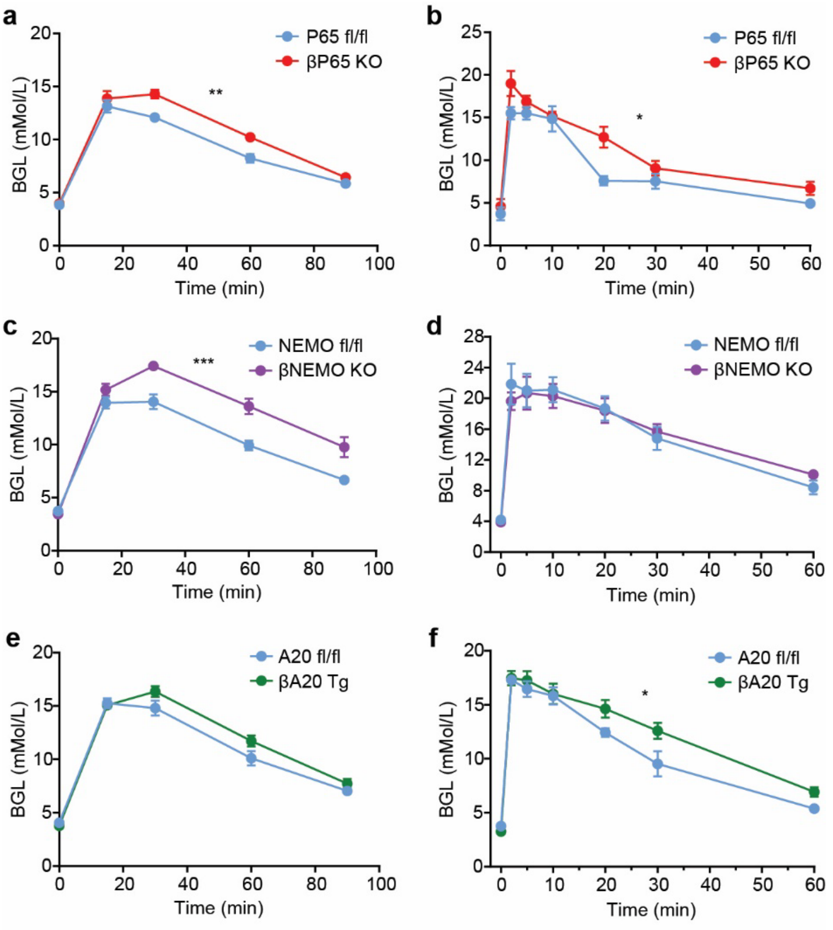

ESM Figure 2. Glucose tolerance test outcomes for male mice.

A) Blood glucose levels (BGL) for 8 week old male mice following an intraperitoneal (i.p. GTT; 2 g/kg) (p65fl/fl *n*=11; βp65KO *n*=11) or **B)** intravenous injection (i.v. GTT; 1 g/kg) of glucose (p65fl/fl *n*=4; βp65KO *n*=5).

C) Blood glucose levels (BGL) for 8 week old male mice following an intraperitoneal (i.p. GTT; 2 g/kg) (NEMOfl/fl *n*=9; βNEMOKO *n*=11) or **D)** intravenous injection (i.v. GTT; 1 g/kg) of glucose (NEMOfl/fl *n*=5; βNEMOKO *n*=5).

E) Blood glucose levels (BGL) for 8 week old male mice following an intraperitoneal (i.p. GTT; 2 g/kg) (A20fl/fl *n*=14; βA20Tg *n*=9) or **F)** intravenous injection (i.v. GTT; 1 g/kg) of glucose (A20fl/fl *n*=4; βA2OTg *n*=7).

Statistical analysis was performed by area under the curve (AUC) analysis, and data shown as the mean +/- s.e.m: *P < 0.05; **P < 0.01; ***P < 0.001.

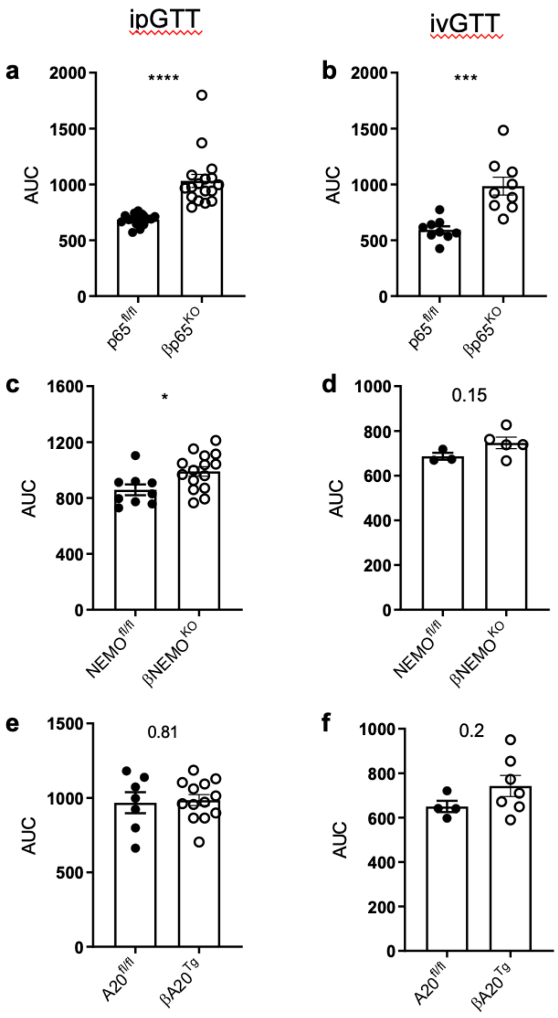

ESM Figure 3. Area under the curve data for GTT studies in NF-kB mutant mice.

Area under the curve data calculated from i.p. GTT and i.v. GTT response for female (a, b) βP65KO; (c, d) βNEMOKO and (e, f) βA20Tg mouse lines respectively. Intraperitoneal (i.p. GTT; 2 g/kg) and intravenous (i.v. GTT; 1 g/kg) injection of 8 week old mice as described in Methods and Results. Statistical analysis was by area under the curve (AUC) analysis, and data shown as the mean +/- s.e.m. *P < 0.05; **P < 0.01; ***P < 0.001 or as indicated.

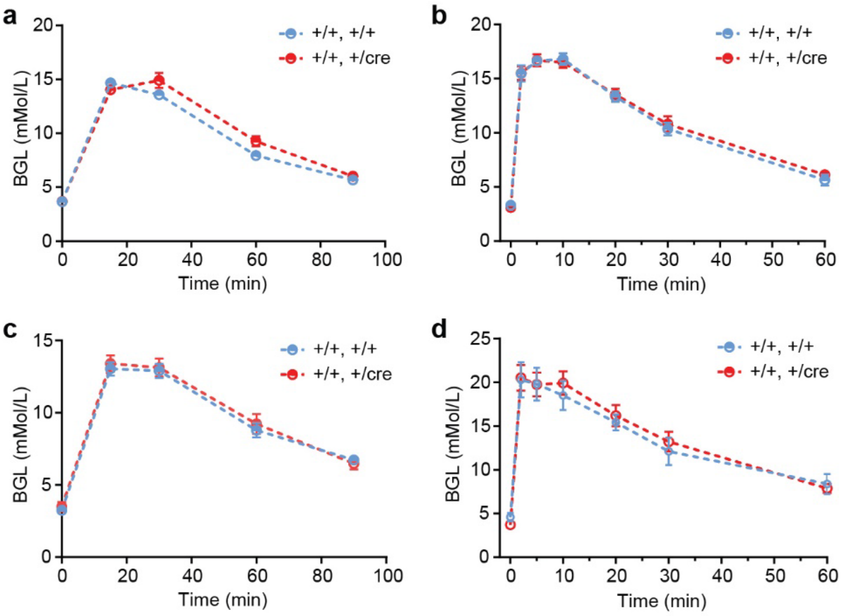

ESM Figure 4. Glucose tolerance test outcomes in non-floxed wild-type and heterozygous Cre male and female mice.

A, C) Blood glucose levels (BGL) following an intraperitoneal (i.p. GTT; 2 g/kg) (+/+, +/+ *n*=15; +/+, +/cre *n*=22) or **B, D)** intravenous injection (i.v. GTT; 1 g/kg) of glucose (+/+, +/+ *n*=8; +/+, +/Cre *n*=14) in 8 week old non-floxed female (A, B) or male (C, D) mice wildtype (+/+, +/+) or heterozygous (+/+, +/cre) for the cre enzyme. Statistical analysis was by area under the curve (AUC) analysis, and data shown as the mean +/- s.e.m.

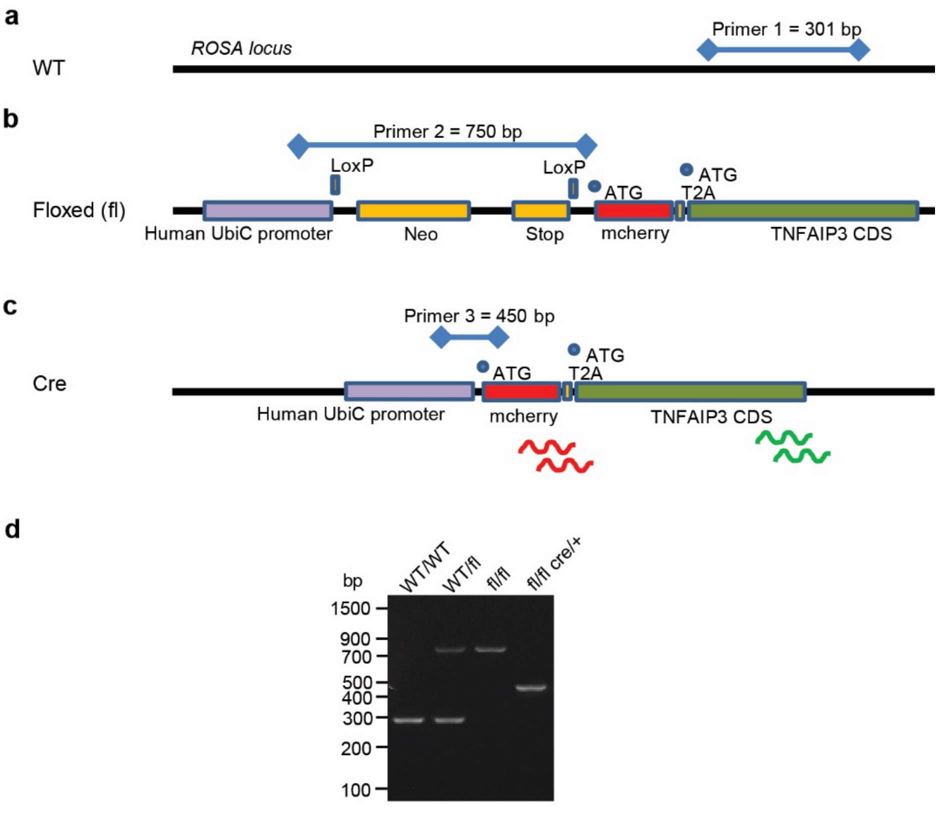

ESM Figure 5. Construct map for A20 transgenic cassette.

A) Schematic of wild-type (WT) and

B) floxed (fl) A20 transgene cassette inserted into the Rosa26 locus of C57/BL6 mice.

C) Schematic of A20 Tg Cassette when Cre enzyme is expressed, resulting in removal of Neomycin (Neo) and stop sequence that are flanked by LoxP flox sites, allowing the transcription of mCherry and *TNFAIP3* genes via the Human UbiC promoter. Primers 1 through 3 are shown with expected size of PCR product (gene sequences in Methods).

D) Electrophoresis gel image of PCR products resulting from PCR analysis of islet DNA extractions using primers shown in A-C.

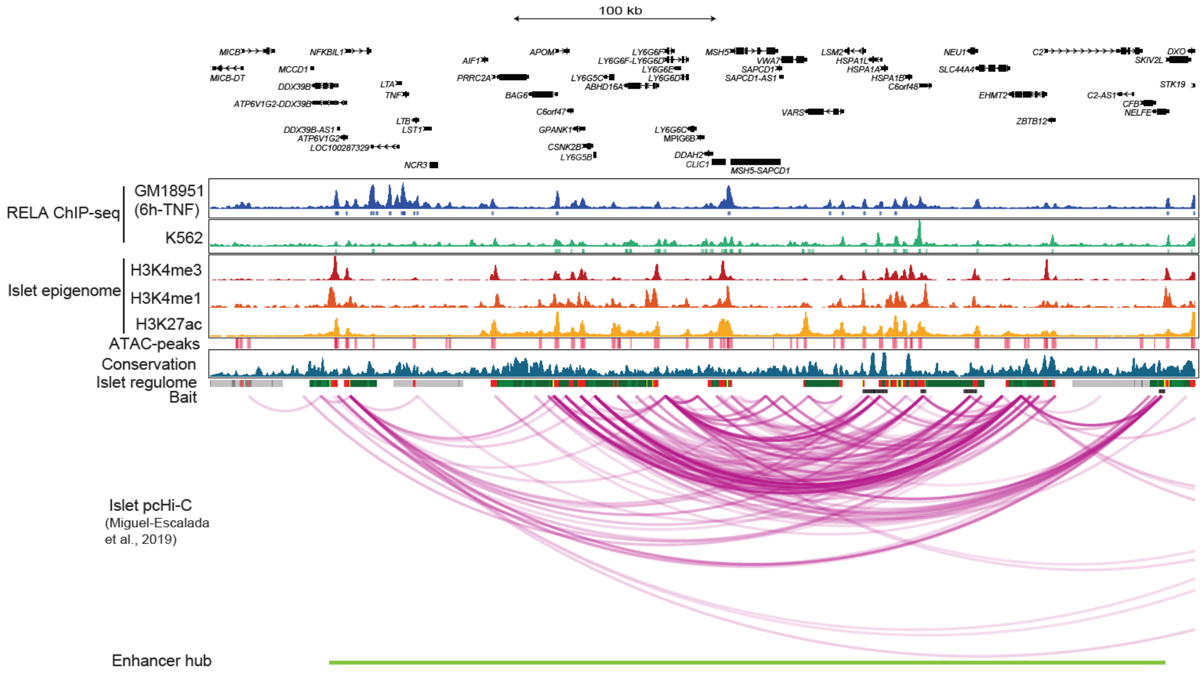

ESM Figure 6. TNF locus forms part of the enhancer hub associated with islet cell functional connectivity and p65 regulation.

Integrative map of the TNF locus, showing p65 ChIP-seq, islet epigenomic annotations and high-confidence pcHi-C interactions within the enhancer hub. p65 ChIP–seq data were obtained from the Encyclopedia of DNA Elements (ENCODE). Islet ATAC-seq peaks and pcHi-C interactions were obtained from the Miguel-Escalada, et al. (1) study. All browser views were generated using WashU Epigenome Browser (2). ChIP, chromatin immunoprecipitation; ATAC, assay for transposase-accessible chromatin; pcHi-C, promoter capture Hi-C.

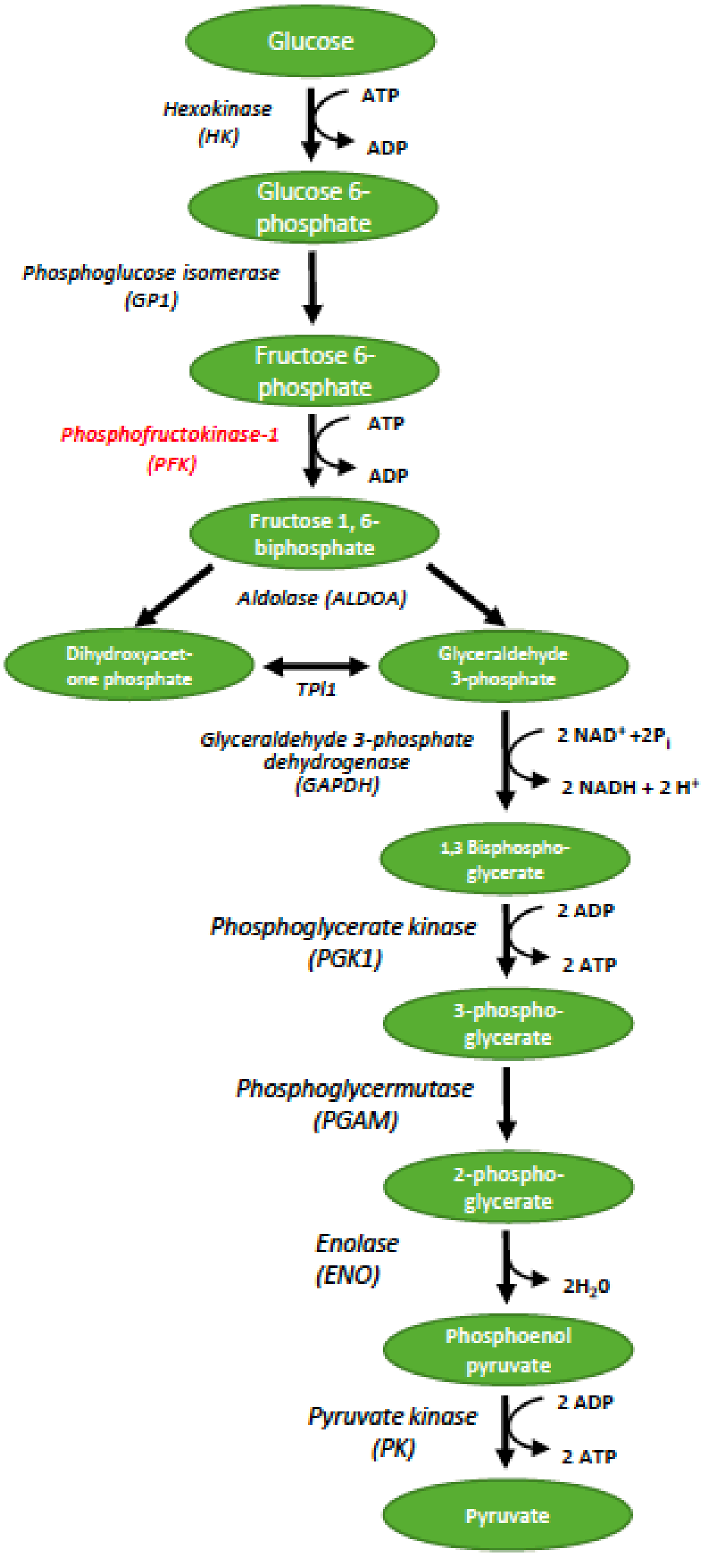

ESM Figure 7. Schematic analysis of glycolysis pathway. Schematic of the major steps in the glycolysis pathway, adapted from *PANTHER Pathways*. Green shade signifies substrates and key metabolic enzymes/genes involved in catalysing each reaction step listed in italics.
